# A Community-based Assessment of Skin Care, Allergies, and Eczema (CASCADE): an atopic dermatitis primary prevention study using emollients—protocol for a randomized controlled trial

**DOI:** 10.1186/s13063-020-4150-5

**Published:** 2020-03-04

**Authors:** Brian Eichner, Le Ann C. Michaels, Kelsey Branca, Katrina Ramsey, Julie Mitchell, Cynthia D. Morris, Lyle J. Fagnan, Rowena J. Dolor, Nancy Elder, David L. Hahn, Donald E. Nease, Jodi Lapidus, Ricardo Cibotti, Julie Block, Eric L. Simpson

**Affiliations:** 1 Duke Primary Care Research Consortium, Duke University, Durham, NC USA; 2 Oregon Rural Practice-based Research Network, Oregon Health & Science University, Portland, OR USA; 3 School of Public Health, Oregon Health & Science University, Portland, OR USA; 4 Wisconsin Research & Education Network, University of Wisconsin-Madison, Madison, WI USA; 5 State Networks of Colorado Ambulatory Partners & Practices, University of Colorado-Denver, Denver, CO USA; 6 National Institute of Arthritis and Musculoskeletal and Skin Diseases, National Institute of Health, Bethesda, MD USA; 7 National Eczema Association, San Rafael, CA USA; 8 Department of Dermatology, Oregon Health & Science University, Portland, OR USA; 9 Oregon Clinical & Translational Research Institute, Oregon Health & Science University, Portland, OR USA

**Keywords:** Atopic dermatitis, Eczema, Pragmatic, Emollient, Moisturizer, Practice-based research network

## Abstract

**Background:**

Atopic dermatitis (AD) is a common, chronic skin disorder often beginning in infancy. Skin barrier dysfunction early in life serves as a central event in the pathogenesis of AD. In infants at high risk of developing AD, preventative application of lipid-rich emollients may reduce the risk of developing AD. This study aims to measure the effectiveness of this intervention in a population not selected for risk via a pragmatic, randomized, physician-blinded trial in the primary care setting.

**Methods:**

Infant–parent dyads are recruited from a primary care practice participating through one of four practice-based research networks in Oregon, Colorado, Wisconsin, and North Carolina. Eligible dyads are randomized to the intervention (daily use of lipid-rich emollient) or the control (no emollient) group (*n* = 625 infants in each) and are followed for 24 months. The primary outcome is the cumulative incidence of physician-diagnosed AD and secondary outcomes include caregiver-reported measures of AD and development of other atopic diseases. Data collection occurs via chart review and surveys, with no study visits required. Data will be analyzed utilizing intention-to-treat principles.

**Discussion:**

AD is a common skin condition in infants that affects quality of life and is associated with the development of other atopic diseases. If a safe intervention, such as application of lipid-rich emollients, in the general population effectively decreases AD prevalence, this could alter the guidance given by providers regarding routine skin care of infants. Because of the pragmatic design, we anticipate that this trial will yield generalizable results.

**Trial registration:**

ClinicalTrials.gov: NCT03409367. Registered on 11 February 2018.

## Background

Atopic dermatitis (AD) is a common, chronic inflammatory skin disorder affecting approximately 13% of children in the United States [1]. The vast majority of cases begin within the first 2 years of life, although onset may occur at any age. Possibly 50% of children with early-onset mild disease outgrow their disease [2]; however, the number of children who experience persistence into adulthood is likely underappreciated [3, 4]. Although the causal mechanisms of the association are not yet fully elucidated, many patients with AD subsequently develop comorbid atopic conditions such as asthma, allergic rhinitis, and food allergies, substantially increasing the disease burden on patients and caregivers [5]. AD negatively impacts a child’s mood, sleep, and activities, and these disruptions can impact a family to a similar degree as having a child with type 1 diabetes [6].

It is increasingly understood that skin barrier dysfunction early in life serves as a central event in the pathogenesis of AD. Studies by Horimukai and colleagues in a Japanese cohort of infants demonstrate that AD begins as subclinical barrier dysfunction prior to visible rash development regardless of the filaggrin gene (*FLG*) status or family history. Skin barrier dysfunction, measured by transepidermal water loss (TEWL), in the first 2 months of life most strongly predicts AD, independent of family history and mutation of the gene encoding the skin barrier protein, filaggrin [7].

Given the role that skin barrier dysfunction likely plays in AD onset, targeting the skin barrier with emollient therapy represents one potential avenue for disease prevention. Emollient therapy represents a conceptually sound approach to the prevention of atopic dermatitis, given that emollient therapy is the cornerstone of flare prevention in children already diagnosed with AD [8, 9]. Lipid-based emollients may prevent the initial flare of the disease and serve a role in primary prevention.

Data within subpopulations have been encouraging. A case–control study from Kenya found that the use of petrolatum on the skin of infants was associated with a reduced risk of AD [10]. A series of studies found that the daily application of Aquaphor ointment or sunflower oil improved skin barrier function and reduced the development of clinical dermatitis in premature neonates [11–17]. The results of pilot trials of patients at high risk for AD demonstrated emollient therapy to be safe and effective in preventing AD [18, 19]. Ohya and colleagues confirmed these preventive effects of emollients in a separate high-risk population of infants [20]. The Barrier Enhancement for Eczema Prevention trial [21], whose protocol was previously published in *Trials,* is studying the application of emollients for AD prevention in a high-risk population for AD across primary and secondary care providers in the United Kingdom. However, it is not known whether emollient therapy serves as a primary prevention strategy in children at normal risk for AD development.

This current large-scale study, titled “A Community-based Assessment of Skin Care, Allergies, and Eczema” (CASCADE), will be the first study of emollient prevention in a population of neonates not selected for risk status. Given that up to 40% of infants who develop AD would not be considered high risk by family history at the time of birth [22], many average-risk individuals could benefit substantially from primary prevention. The primary prevention of AD with emollients in the general population has not yet been studied and it is not known whether it is feasible for the general population to apply emollients daily over the first 2 years of life.

### Objectives

The primary objective of the CASCADE study is to assess whether the prevalence of AD in the first 2 years of life can be diminished by daily application of emollients by caregivers. Further, the study will assess the effects of emollient therapy on AD symptom burden and the development of allergic comorbidities.

## Methods

The study team engaged with three planning groups to develop the protocol: a Community Advisory Committee, a Protocol Advisory Committee, and directors of the Meta-network Learning And Research Center (Meta-LARC) consortium of practice-based research networks (PBRNs) from five states. These committees gave input on the setting, design, and intervention elements and cooperated in developing study-related education, adherence measures, incentives, survey questions, timing of enrollment, and outcome measures. The Protocol Advisory Committee included members with epidemiology, clinical trials, data coordination, pediatrics, and statistical expertise. The Community Advisory Committee comprised clinicians in family medicine, pediatrics, and dermatology, primary care administrators, patient advocacy groups, and patients. Each of the five US-based PBRNs participating in the Meta-LARC consortium involved a director and research coordinator, with some PBRNs contributing a practice facilitator to participate in monthly planning meetings to provide input on feasibility and study implementation.

PBRNs allow primary care clinics to participate meaningfully in multisite research generalizable to clinical practice while limiting logistical and feasibility obstacles. The Meta-LARC consortium includes 4000 primary care clinicians in 1000 practices who care for 4 million patients in rural, urban, and underserved communities. As part of the protocol development, a planning and feasibility study (U34 AR065739 01A1) was undertaken in 10 Meta-LARC clinics in five states that involved surveying parents of 652 children regarding the presence of an AD diagnosis. Key data generated from the planning project were the confirmation of high disease prevalence in primary care offices (24% by year 2) [23] and recognition that recommending emollients may not be as effective as physically providing emollients, given that 38% of parents still reported using a watery lotion even after receiving a brochure regarding the potential benefit of thicker emollients**.**

### Design

**Fig. 1:**
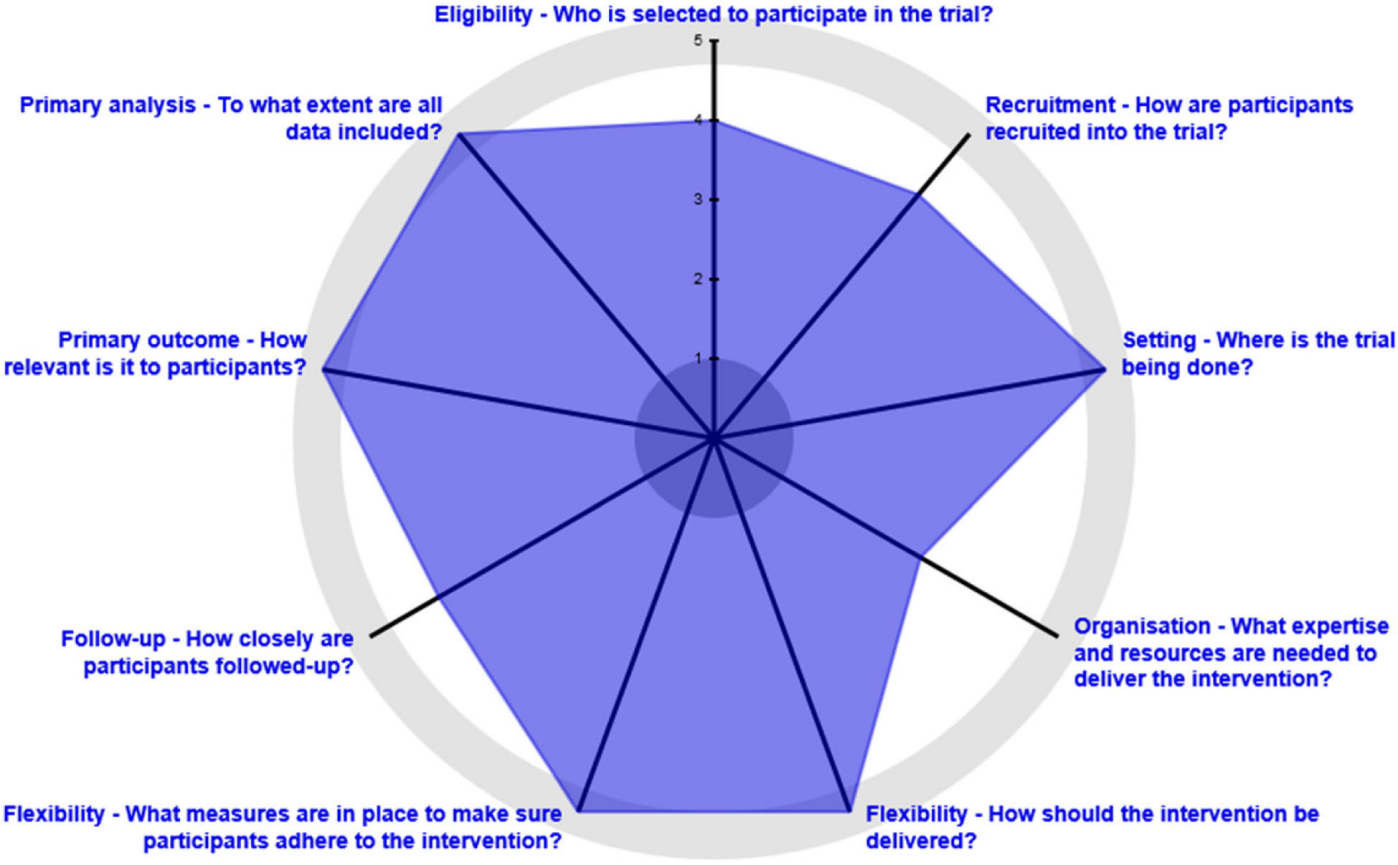
Pragmatic Explanatory Continuum Indicator Summary (PRECIS-2) wheel for aspects of the pragmatic trial. The investigators have quantified each of the nine PRECIS-2 domains along the “explanatory–pragmatic continuum”, where 1 = most explanatory and 5 = most pragmatic. Generally, pragmatic trials correspond with “real-world effectiveness trials” and explanatory trials correspond with “efficacy/mechanistic trials”

The CASCADE study is a randomized, multisite, single-blind pragmatic trial comparing the efficacy of the daily use of emollients versus usual care (no routine emollient use) to prevent AD from early infancy to 24 months. The trial plans to recruit 1250 infant–parent dyads to be randomized to each arm in a 1:1 ratio (625 per arm), stratifying on risk. Outcome data will be collected via chart review by blinded PBRN research coordinators and via electronic questionnaires filled out by parents at baseline, 12 months, and 24 months. The pragmatic aspects of this trial include broad inclusion of a general population recruited through community-based primary care practices, no scheduled research visits, minimal adherence monitoring, and participants who are provided a choice within the intervention arm. Figure 1 demonstrates the degree of pragmatism of the CASCADE study using the standardized PRECIS-2 tool [24]. While the protocol design fulfills many pragmatic research design criteria, its biggest weakness is in the organization category, given that the study provides emollients for the patients randomized to the intervention arm and, outside the research setting, families have to purchase emollients out of pocket.

### Setting

The CASCADE study will recruit families whose children attend one of 25 US primary care clinics (pediatric and family medicine) in Oregon (*n* = 10 clinics), Wisconsin (*n* = 5), Colorado (n = 5), and North Carolina (n = 5). All clinics are voluntary members of regional PBRNs. These four PBRNs are part of a larger meta-network of PBRNs established by the Agency for Healthcare Research and Quality as a Center of Excellence for primary care research. Meta-LARC was established to develop an infrastructure to rapidly plan and implement multicenter research studies in primary care and has conducted large-scale studies in self-management support and advance care planning. The PBRNs within Meta-LARC reach over 750 practices in the United States and 400 practices in Canada, serving a combined 3.8 million patients. For the CASCADE study, the participating Meta-LARC PBRNs and their host academic institutions include the Oregon Rural Practice-based Research Network (Oregon Health & Science University), the State Networks of Colorado Ambulatory Practices & Partners (University of Colorado), the Wisconsin Research & Education Network (University of Wisconsin), and the Duke Primary Care Research Consortium (Duke University, North Carolina).

### Study oversight and regulatory considerations

**Fig. 2:**
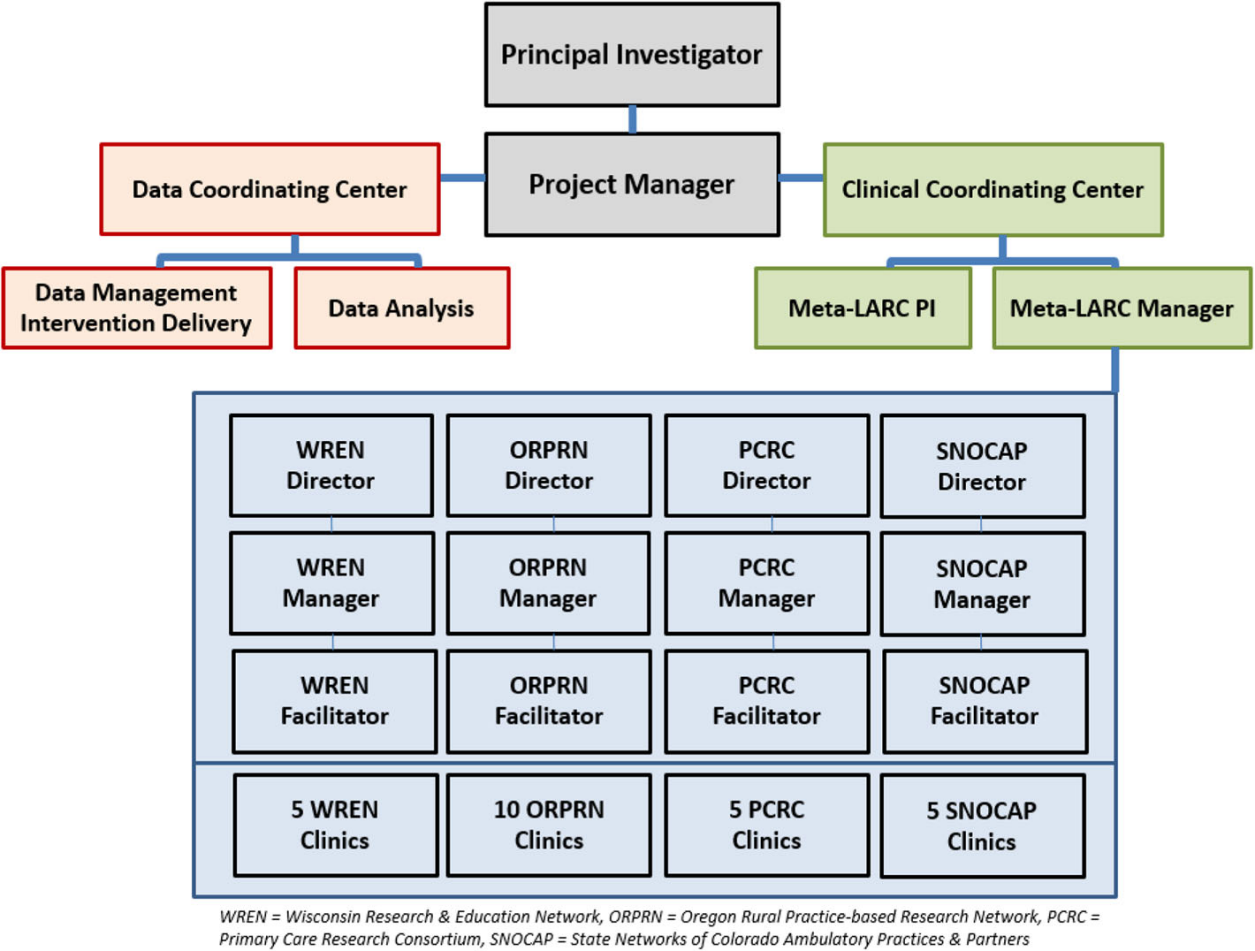
Organizational chart for the CASCADE study. CASCADE A Community-based Assessment of Skin Care, Allergies, and Eczema, Meta-LARC Meta-network Learning And Research Center, ORPRN Oregon Rural Practice-based Research Network, PCRC Primary Care Research Consortium, PI Principal Investigator, SNOCAP State Networks of Colorado Ambulatory Practices & Partners, WREN Wisconsin Research & Education Network

Trial management will be conducted through a Clinical Coordinating Center (CCC) and a Data Coordinating Center (DCC), both having oversight from the study PI (Fig. 2).

The CCC utilized the Clinical and Translational Science Award (CTSA) Trial Innovation Network to identify a single Institutional Review Board (sIRB) through the University of Utah to oversee human subjects’ involvement. The Oregon Health & Science University (OHSU) site was established as the clinical coordinating center and each PBRN completed local human research protection reviews with each individual IRB relying on the University of Utah. Ongoing protocol amendments and annual continuing reviews will be reported to the University of Utah.

KAI Research, a vendor utilized by the National Institutes of Health, established an independent Data and Safety Monitoring Board (DSMB) and will facilitate meetings of the DSMB every 6 months.

### Participants

Infant–parent dyads will be enrolled and randomized when the infants are no older than 9 weeks. The parent must be at least 18 years old at the time of consent and speak, read, and write in English or Spanish. The parent must have a valid email address and reliable access to the Internet and the infant must be a patient at a participating clinic affiliated with Meta-LARC at the time of enrollment. Infants will not be included in the study if they are born at less than 25 weeks gestational age or have an extremely low birth weight (less than 1000 g), have an existing diagnosis of eczema, have a known immunodeficiency genetic syndrome, have a known adverse reaction to petrolatum-based emollients, or have a sibling enrolled in the study.

### Recruitment

Infant–parent dyads are invited to enroll during a regularly scheduled visit to their participating primary care clinic prior to 9 weeks of age. All providers whose patients may participate have watched a mandatory eczema diagnosis video recorded by a dermatologist. The parent or guardian accesses the electronic REDCap-based questionnaires for screening, consenting, and enrollment. REDCap (Research Electronic Data Capture) is a secure, web-based application designed to support data capture for research studies [25]. The study questionnaires can be accessed using an electronic tablet in the office or families can access the link from any other web-enabled device. Due to the regulatory burden for small primary care clinics, office staff are not engaged in research. Thus, participants are only offered the study recruitment materials (electronic tablet or rack card, postcard or flyer with the Internet link) by clinic staff. During the consenting process, potential participants are provided the opportunity to speak to a central study staff member if they have questions about the study or consent form. The electronic consenting process meets IRB requirements and participating families receive copies of their consent via email and mailed to their home with a study welcome packet.

### Intervention and assignment of intervention

This is a two-arm study. Randomization to the intervention group versus the control group (1:1 ratio) occurs after enrollment via the central DCC and uses blocked randomization in groups of four. The randomization list is generated by a study statistician using a computer-based random generator and is loaded into REDCap. The REDCap randomization plug-in automatically assigns the next available unused randomization ID that matches by site and family history. Once an individual is randomized, the system does not allow reuse of a randomization ID. The study statistician is responsible for generating the randomization codes and periodically reviewing the randomization to ensure that the system is working as it should in assigning treatments. Approximately 625 (half) of the infant–parent dyads will be assigned to the intervention arm, in which they will receive emollient by mail and be instructed to apply it daily for 24 months. Their physicians are blinded to enrollment status and to which arm the participants are assigned. Research staff abstracting data from the infant’s health record and the principal investigator are also blinded. There will be no special criteria for discontinuing or modifying allocated interventions. The parents can select from five potential lipid-rich emollients (CeraVe Healing Ointment, Cetaphil cream, Vanicream, CeraVe Cream, and petrolatum/Vaseline) that have been shown to improve skin barrier function. Emollients were selected based on ingredients (high lipid content and lack of potential sensitizers), clinical and skin barrier data supporting products of similar formulation, regulatory/feasibility considerations, and recommendations by pediatric dermatologists from the Pediatric Dermatology Research Alliance. The selected emollient will be shipped by the DCC within 72 h of randomization to the participants’ home. Both groups will receive identical recommendations for general bathing and cleansing. Parents in the control group will be asked to refrain from regular emollient use (Natural Skin Group), but may use them on an as-needed basis if dry skin develops. Participants in both groups receive identical skin care advice including recommendations for the frequency of bathing. The intervention group (Everyday Moisturizer Group) will be instructed to apply full body emollient to all body surfaces daily, with the scalp and diaper areas being optional. Quarterly touchpoints by research staff will help maintain intervention adherence, and this is within the realm of the frequency of visits to the pediatric office for a well child in this age range, thus minimally interfering with the protocol’s pragmatic design. The suppliers for the emollients are as follows: CeraVe/L'Oreal USA(10 Hudson Yards, New York, NY 10001); Cetaphil/Galderama Laboratories, L.P.(14501 N. Freeway, Fort Worth, TX 76177); Vanicream/Pharmaceutical Specialties, Inc.(1620 Industrial Dr. NW, Rochester, MN 55901); Vaseline(Unilever US, Inc, 800 Sylvan Avenue, Englewood Cliffs, NJ 07632).

### Outcome measures

The primary outcome measure is the cumulative incidence of AD at age 24 months (+ 3-month window) as recorded by the participants’ usual clinician. During monthly monitoring visits, PBRN coordinators remind clinicians of the importance of accurate documentation of the presence of AD in the medical record for all of their patients irrespective of CASCADE enrollment. PBRN coordinators trained in chart review for project outcomes will be responsible for reviewing each subject’s medical records throughout their dates of enrollment.

Secondary outcome measures can be broadly grouped into several categories: alternative AD criteria, such as parental report of clinician-diagnosed eczema and as diagnosed by the Children’s Eczema Questionnaire [26]; symptom burden such as sleep disruption, use of topical corticosteroids (over the counter or prescribed), and Global Health Status using one question from the Patient-Reported Outcomes Measurement Information System (PROMIS) Pediatric Global Health checklist (PGH-7); and comorbid conditions such as presence of food allergy symptoms, food allergy clinical diagnosis (by skin prick or IgE testing), and asthma risk via a truncated version of the Asthma Predictive Index (mAPI). Due to budgetary constraints, prospective skin prick testing will not be performed.

Additionally, for participants with AD, secondary outcome measures will be the time of onset to AD (by report and by diagnosis in chart), AD symptom severity (as measured by the Patient-Oriented Eczema Measure, or POEM, instrument), parent-reported global severity of eczema assessment, and the Infant Dermatology Quality of Life Instrument (IDQOL).

### Data collection

**Fig. 3:**
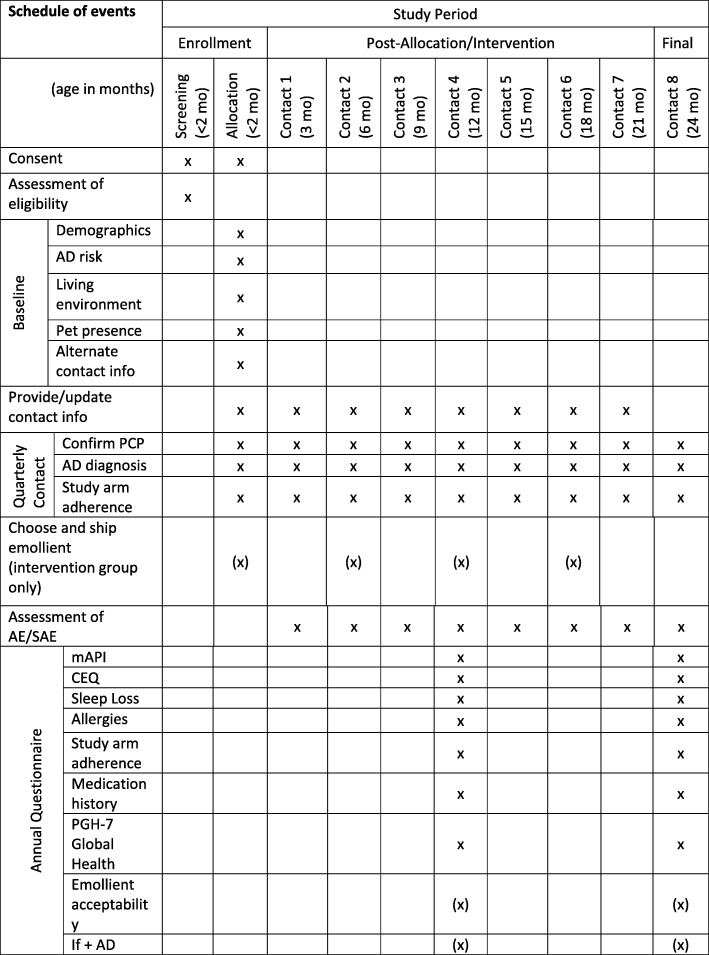
Standard Protocol Items: Recomvhmendations for Interventional Trials (SPIRIT) checklist including milestones of enrollment and data collection during the CASCADE study. AD atopic dermatitis, AE adverse events, CASCADE A Community-based Assessment of Skin Care, Allergies, and Eczema, CEQ Children’s Eczema Questionnaire, mAPI modified Asthma Predictive Index, mo months, PGH-7 Pediatric Global Health checklist, SAE serious adverse events, PCP Primary Care Provider

Data to be collected and time points are summarized in Fig. 3. Of note, the parent–infant dyads will not be required to have any in-person visits with the study staff. Subject-supplied data collection will occur via electronic surveys at baseline, 12 months, and 24 months with brief surveys conducted quarterly for adverse event elicitation and contact information updates. All survey invitations are initiated via REDCap and participants record their survey responses directly into the database. Chart abstraction will be performed by intervention-blinded research coordinators at 27 months and will include the collection of data such as clinician diagnosis of AD, adverse events (AEs), and clinician diagnosis of allergies during the study period. This trial does not involve collecting biological specimens for storage. Participant retention will be enforced utilizing text messages and telephone calls for non-responders to questionnaires.

### Data management

The DCC at OHSU has responsibility for maintaining the integrity and completeness of data collection, for storage of study data to protect confidentiality, and for managing data quality through continuous assessment. All data are stored in a REDCap database and entered directly by study participants, and by study staff for data collection from the medical record. The DCC is responsible for enrollment and randomization, ensuring patient consent documentation, initiating participant reminders, disseminating surveys to participants, and monitoring their completion. The DCC also receives and reviews skin product-related AEs that are reported on quarterly surveys.

### Analysis

We will analyze all primary and secondary outcomes using intention-to-treat principles as our main approach. Additional per-protocol analyses will include parent-reported frequency of emollient use.

Subjects who are lost to follow-up or withdraw from the study will not be replaced but their data will be included in the analysis under multiple imputation. Participants who withdraw consent will not have their data evaluated beyond the dates of this occurrence. For those who do not adhere to the intervention, their data will be analyzed utilizing intention-to-treat principles and analyzed according to their randomized group.

Our primary hypothesis is that the intervention will result in significantly lower incidence of provider-diagnosed atopic dermatitis by age 24 months. We will test the hypothesis that the intervention:control population ratio (relative risk) is significantly smaller than 1 using a two-sided test of the coefficient for the intervention group in a log-binomial regression model [27] that also includes the stratification variables to minimize bias [28]. Log-binomial regression is similar to logistic regression but provides estimates of relative risk rather than odds ratios.

Secondary measures include alternative diagnostic definitions of atopic dermatitis (parent report [29], the Children’s Eczema Questionnaire (CEQ) [30]), proportion of patients with asthma (via mAPI) [31]) and prevalence of provider-diagnosed parent-reported food allergy [32]. These binary outcomes will be evaluated with similar log-binomial models.

Non-binary secondary outcomes will be evaluated in manners consistent with their outcome types. Differences in the 5-point validated global health question of the PROMIS-PGH7 [33] will be measured with a chi-squared test. Outcomes with continuous variables such as the POEM [34] and the IDQOL [35] will be evaluated by histograms for obvious lack of symmetry, and between-group differences will be estimated using linear regression similar to the log-binomial models described earlier. Differences in the age of onset of AD will be evaluated using the Kaplan–Meier estimator for both the age at first provider-recorded date of diagnosis and the parental report of eczema age of onset. Prescription topical medication and over-the-counter hydrocortisone usage will be measured in separate analyses of all children and only those children with AD. Mean days of sleep disruption will be tested with a Poisson model as it will be treated as a count variable.

As already mentioned, randomization will be stratified by family history of AD in a first-degree relative. This will also be evaluated as a potential difference in treatment effect, as will factors such as dry climate, presence of pets (cats and dogs) in the home, sex, race, and ethnicity.

One interim analysis for efficacy is planned for when half of the projected sample in each allocation group have completed follow-up. The trial will stop if differences between the intervention and control groups are significant at a nominal α = 0.003 for the interim analysis. For the final analysis, tests will be evaluated at α = 0.047, maintaining an overall type I error rate of 0.05. This approach requires strong evidence for early stopping but maintains a critical value for the final analysis that is close to what it would have been without interim analysis.

### Sample size

We anticipate a cumulative incidence of eczema of 24% at age 24 months without intervention [23]. To estimate at least 30% relative reduction in atopic dermatitis, we require 1044 dyads (522 per group) to achieve 80% power for a two-tailed test at the 0.05 level of significance with one midpoint analysis. A pilot study in a population at a high risk of developing atopic dermatitis demonstrated a 50% relative reduction in AD [18], and thus powering the study for a 30% relative reduction was determined to be a conservative estimate. Assuming approximately 20% loss to follow-up, we plan to enroll 1250 babies or 625 in each arm.

### Monitoring

Safety monitoring will entail the evaluation of adverse events, serious adverse events, and unanticipated problems as reported to a Data and Safety Monitoring Board (DSMB) established by the NIAMS. The treatment protocol and data collection procedures meet the definition of minimal risk. Reportable adverse events in the study are all skin product-related. All serious adverse events will be collected. The collection of adverse events and serious adverse events occur via participant report to the DCC via the quarterly surveys embedded in the study protocol and participant report to the CCC via telephone call or email. The DCC audits the trial on a daily basis, running reports that include participant consent process completion, intervention integrity, survey responses, and adverse event monitoring. The DSMB and DCC are independent from the CCC. Skin product-related adverse events identified during chart abstraction by the regional research coordinators will also be collected. Adverse events assessed to be related, severe, and unexpected will be reported as unanticipated problems. There is no anticipated harm for trial participation and thus there is no provision for post-trial care outside the usual skin care recommendations by the participant’s primary care provider. Three unblinded sub-investigators will assess AEs for severity, relatedness, and expectedness.

### Dissemination

We plan to disseminate the results of this large, pragmatic randomized controlled trial to various segments of the population given the broad, relatable interest associated with a disease of such high prevalence. The results will be submitted for publication to an appropriate medical journal and for presentation at relevant society meetings of primary care physicians and dermatologists. The results will be shared promptly within each PBRN, with all of the affiliated recruitment clinics, and with the research participants. Additionally, we will share our results with community agencies, particularly those who focus on infant health education, and third-party payers, given that the possibility of prevention of eczema with lipid-rich emollients could have implications on their potential coverage by insurance companies, particularly if subsequent analysis found this to be a cost-effective approach.

## Discussion

This pragmatic randomized controlled trial will evaluate whether the cumulative incidence of AD, its morbidity, and associated comorbidities are modified by the regular use of lipid-rich emollients starting in the neonatal period through age 24 months. This study will involve a large number of infant–parent dyads across a network of community-based clinics in the United States that are heterogeneous in geography, climate, and practitioner setting. The question being addressed is of high impact: how should we care for the skin of our newborns? The results of this study, especially given its pragmatic design, could immediately inform skin care guidelines for the majority of US newborns.

The highly pragmatic elements of this study make it novel among studies in dermatology; the primary care community setting for the research adds additional novelty. The study interventions and procedures can all be carried out simply in their real-world home environment with follow-up outcome measurement occurring via electronic surveys and routine clinical practice. The intervention is not burdensome to clinic staff and thus would be reproducible in the general clinic setting. The intervention itself has minimal risk, making it appealing to a broader group of families in primary care whose children are, at baseline, typically healthy and perhaps hesitant to expose their infants to interventions. Given the absence of dedicated research visits in combination with the parental choice of emollient, this closely mimics a more realistic clinical setting than typical controlled trials at academic centers can achieve.

Another novel aspect of this study is the study population. All previous studies evaluating emollients for AD prevention have focused on patients who have a high risk of developing AD determined by family history. Targeting a high-risk population utilizing family history, while likely improving efficiency, would miss a significant proportion of infants who will go on to develop AD [22]. Due to the high prevalence and morbidity of AD, studies such as this are necessary to guide skin care recommendations given to all parents, especially given that the majority of parents are already applying skin care products to their children [36, 37].

Given the generalizability of the results from this trial, positive or negative, we are examining the effectiveness, rather than the mere efficacy, of emollients to prevent or temper the effects of atopic dermatitis in infants. We are also optimistic that this trial can serve as a model for the collaboration between primary care PBRNs and specialty medicine. There is a necessarily blurred line between specialty care and primary care in the management of common illnesses with broad ranges of severity and the expertise of both types of researchers and clinicians are needed in this type of trial. PBRNs provide an underutilized “real-world” laboratory for pragmatic clinical trials. There are multiple examples of medical conditions in which research benefits using this collaborative model may have utility in the future. This technique was successfully employed in the planning portion of the CASCADE study. This trial builds on the CASCADE planning phase success attributable to respectful communication and joint problem-solving to overcome barriers. We hope to include any lessons learned in the dissemination of the results of this trial and to inform future study designs.

### Trial status

At the time of this submission, patient recruitment and enrollment, which began July 2018, had started at all of the practice sites. Enrollment is anticipated to continue through October 2020 with follow-up continuing through January 2023. Protocol version 9.0, February 18, 2019.

## Data Availability

Data are participant-reported and collected for research purposes. Public release of clinical trial data will follow the NIAMS and NIH guidelines. The datasets used and/or analyzed during the current study are available from the corresponding author on reasonable request.

## Abbreviations

AD: Atopic dermatitis
CASCADE: A Community-based Assessment of Skin Care, Allergies, and Eczema
CCC: Clinical Coordinating Center
CEQ: Children’s Eczema Questionnaire
CTSA: Clinical and Translational Science Award
DCC: Data Coordinating Center
DSMB: Data and Safety Monitoring Board
*FLG*: Filaggrin gene
mAPI: Modified Asthma Predictive Index
Meta-LARC: Meta-network Learning And Research Center
OHSU: Oregon Health & Science University
PBRN: Practice-based research network
PCP: Primary Care Provider
PGH-7: Pediatric Global Health checklist
PI: Principal Investigator
POEM: Patient-Oriented Eczema Measure
PRECIS-2: Pragmatic Explanatory Continuum Indicator Summary
PROMIS: Patient-Reported Outcomes Measurement Information System
REDCap: Research Electronic Data Capture
sIRB: Single Institutional Review Board
TEWL: Transepidermal water loss
